# Incorporating coherent multiple scattering into modelling of small-*q* scattering data

**DOI:** 10.1107/S1600576726005066

**Published:** 2026-07-22

**Authors:** Henrich Frielinghaus

**Affiliations:** a Forschungszentrum Jülich GmbH, Jülich Centre for Neutron Science JCNS at MLZ, 85748 Garching, Germany; Lund University, Sweden

**Keywords:** coherent multiple scattering, Mie scattering, ultra-small-angle neutron scattering, ultra-small-angle X-ray scattering, small-angle light scattering

## Abstract

In a recent paper [Frielinghaus & Gommes (2025), *J. Appl. Cryst.***58**, 1553–1570] the appearance of coherent multiple scattering was discussed, whereby surface scattering takes over against the classical bulk scattering. This effect has been overlooked in the past and happens in a transition region between the classical bulk scattering that involves the first-order Born approximation and the quantum mechanical limit where the projected shadow of the structure is observed. This paper discusses a new example emerging from small-angle light scattering and describes it with surface scattering from the Teubner–Strey model that is also used to describe porous materials.

## Abstract 2

A new example for coherent multiple scattering is presented that confirms the Teubner–Strey model with its surface scattering for porous materials.

## 1. Introduction

The classical bulk scattering is usually used to describe and model small-angle scattering data. For this, the first-order Born approximation is used. The amplitudes of the scattered waves emerge from a Fourier transform of the real-space structure, *i.e.* of the scattering length density (SLD) profile inside the sample. The parameter of the Fourier transform is the scattering vector **q**, which connects to the experimental setting with the scattering angle θ and the probe wavelength λ as 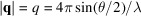. It easily can be generalized to vectorial magnitudes, but for isotropic scattering it is often kept at this level. The detectors only detect intensities, and so the modulus of the amplitude needs to be squared. That corresponds to a loss of phase information [*i.e.* the sign (+/−) of the amplitude is lost] and so the real-space structure can only be reconstructed with some assumptions [the software packages *DENFERT* (Koutsioubas & Pérez, 2013 ▸; Koutsioubas *et al.*, 2016 ▸) and *DAMMIF* from *ATSAS* (Franke & Svergun, 2009 ▸; Franke *et al.*, 2025 ▸) provide this], while the real-space correlation function *p*(*r*) can be obtained quite robustly (also provided by the above-mentioned software packages). Further scattering profile modelling based on the first-order Born approximation involves either simple morphologies (Pedersen, 1997 ▸) or statistical physics descriptions of the structure that can be Fourier transformed.

In our previous paper (Frielinghaus & Gommes, 2025 ▸) we discussed more exact approaches that describe small-angle scattering experiments to a higher precision and introduced a new condition where surface instead of bulk scattering is observed. The former comes into play for small-angle light scattering (SALS), ultra-small-angle neutron scattering (USANS), ultra-small-angle X-ray scattering (USAXS) and very small angle neutron scattering (VSANS), rather than in classical small-angle neutron and X-ray scattering (SANS/SAXS). The critical parameter 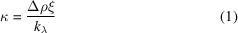
was derived (Frielinghaus & Gommes, 2025 ▸; Berk & Hardman-Rhyne, 1986 ▸), where Δρ is the SLD difference between a colloid (or particle) and the surrounding environment (solvent or vacuum/air, for example). For neutrons there are tables (Sears, 1992 ▸) and web portals (US National Institute of Standards; https://www.ncnr.nist.gov/resources/activation/) that calculate the SLD. The web portal also includes the SLD for SAXS that refers to the density of electrons multiplied by the classical electron radius (Thomson length). In the latter case, the SLD is energy dependent and often carries an imaginary part for strong absorption. In SALS the SLD difference is given by Δρ = −(2π*n*_0_/λ^2^)(d*n*/dϕ) for solutions and by Δρ = π(1 − *n*^2^)/λ^2^ for solids against a vacuum. Here, the refractive index *n* also appears in the derivative of the volume fraction of sample material ϕ (colloid) in the solvent connected to the index 0 in *n*_0_. The light wavelength is denoted λ and in practice is about a couple of hundred nanometres. The correlation length ξ is connected to the sample structure. For spheres with a radius *R* one finds ξ = 4*R*/3. Generally, the correlation length can be calculated from 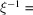

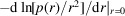
. The momentum of the incoming probe *k*_λ_ = 2π/λ is tightly connected to the wavelength λ.

The new parameter κ classifies the validity of different approaches to modelling the scattering profile. For |κ| ≲ 0.1 the classical first-order Born approximation holds. This means that inside a typical volume of approximately ξ^3^ the probe scattering is described by the usual approach (more exactly, the minimum volume of the correlation length ξ and the coherence volume). Only if the sample thickness is too large may several independent scattering events happen along the probe flight path inside the sample. That kind of (incoherent) multiple scattering is described in a previous article [Jaksch *et al.* (2021 ▸) and references therein].

For larger |κ| of up to 10 or even 100, coherent multiple scattering appears, which leads to scattering from the colloid surfaces only, *i.e.* within the volume ξ^3^. This approach was obtained by comparisons between the full Born expansion and an analytical (quantum mechanical) solution for spherical colloids (Frielinghaus & Gommes, 2025 ▸). The latter solution is also known in light scattering from the Mie theory (Mie, 1908 ▸). Modelling of surface scattering (emerging from a simple Fourier transform) is still within the scope of most theoretical models (Ruckdeschel *et al.*, 2016 ▸; Wuttke, 2021 ▸; Volkov & Svergun, 2003 ▸). So, for simple colloidal shapes (Pedersen, 1997 ▸) the surface scattering is analytical. For porous materials, the author recently published a connection between bulk and surface scattering functions (Frielinghaus, 2026 ▸).

One other important example uses Gaussian random fields to model various scattering functions (Gommes *et al.*, 2021 ▸). For fractal structures with a power law *q*^−α^, there is a simple transition to another power law *q*^α−6^ towards lower *q* [see reports investigating scattering by office paper (Frielinghaus & Gommes, 2025 ▸; Ji *et al.*, 2022 ▸)]. This transition corresponds to surface fractals for the bulk scattering and to mass fractals for surface scattering.

This behaviour was already speculated by Mildner & Hall (1986 ▸). The final limit to the quantum mechanical scattering is – so far – not given by a strict transition value for |κ|. However, most practical examples will not go much further than about |κ| ≃ 100.

**Figure 1:**
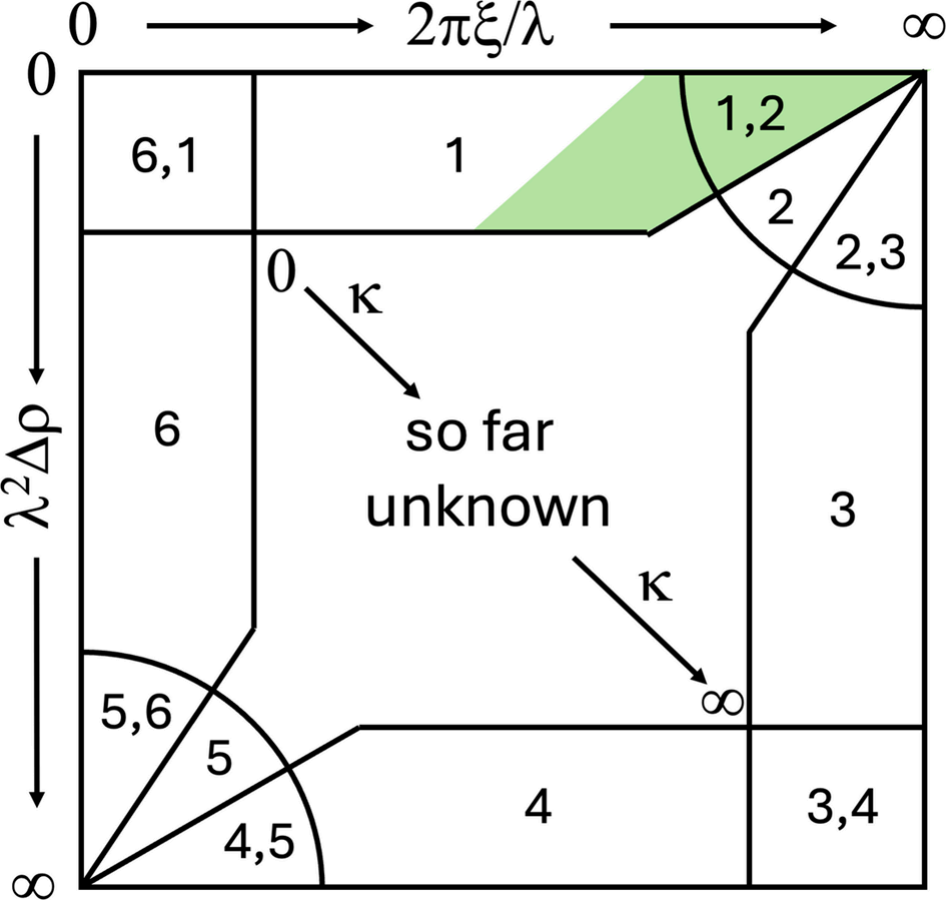
The parameter scheme of van de Hulst (1957 ▸; see p. 132, Fig. 20), adapted for this work. The horizontal axis is connected to the typical phase shift, *i.e.* the typical path difference given by the colloidal size and the wavelength. The vertical axis scales with the dimensionless contrast, *i.e.* the SLD difference scaled with the wavelength, which in optics is directly connected to the refractive index difference *n* − 1. The theory classification is indicated by areas as follows: (1) Rayleigh–Gans or first-order Born approximation, (2) anomalous diffraction or WKB approximation, (3) large spheres, (4) total reflector, (5) optical resonance and (6) Rayleigh scattering. Transition regions are indicated by two numbers. The green area indicates the transition range between (1) and (2) where coherent multiple scattering dominates. Under these circumstances, surface scattering dominates, *i.e.* the scattering emerges from the surface of the structure.

The case of coherent multiple scattering has to be seen in context with the anomalous surface reflection described by Yoneda and Vineyard (Yoneda, 1963 ▸; Guentert, 1965 ▸; Vineyard, 1982 ▸). It is the resonances at surfaces at shallow incident angles that highlight the surfaces as such. In reflectivity measurements, an additional peak is observed close to the horizon of the surface. In small-angle scattering experiments, only surface scattering appears and it takes over for the observed scattering profile. The classification of this transition region in SALS was not treated exhaustively in standard texts [see *e.g.* van de Hulst (1957 ▸), p. 132, Fig. 20]. An adapted version of this figure is presented in Fig. 1 ▸, where the green colour indicates the transition region (1,2) where coherent multiple scattering dominates.

The missing point is the scattering law itself which was – to the best of my knowledge – not described before, although it simply emerges from the surface structure.

When looking at a wide *q* scan employing several small-angle scattering methods (*e.g.* SALS, USANS/USAXS, VSANS and classical SANS/SAXS) to characterize a hierarchical structure with many length scales, one can identify a critical *k*_c_ = 4|Δρ|λ that divides the full *q* axis into two regions. For *q* < *k*_c_ one finds the larger structures that only display their surface, while for or *q* > *k*_c_ the smaller structures show classical bulk properties that most experimentalists are trained to work with. The full connection of the concepts is published by Frielinghaus & Gommes (2025 ▸). The transition to the quantum mechanical limit that is possible in theory will not be observed by most small-angle scattering experiments. From van de Hulst (1957 ▸), the transition is characterized by the scaling of 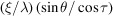 ≃ κ ≃ 10 to 100, a condition for the scattering of a sphere’s apex. The small typical scattering angle θ ≃ *k*_c_λ scales with the critical scattering angle *k*_c_, and the small typical incident angle τ causes the cosine to be approximately unity. The typical values of κ to transition to the quantum mechanical limit of 10 to 100 result from exact solutions of the sphere scattering (Frielinghaus & Gommes, 2025 ▸), where considerable deviations from simple surface scattering (coherent multiple scattering) become visible. The transition range from region (1) to region (2) is indicated by the green area in Fig. 1 ▸. This transition corresponds to the series of (1) the first-order Born approximation to coherent multiple scattering (green) to (2) anomalous diffraction which is also described by the WKB (Wentzel/Kramers/Brillouin) theory (Shepelevich *et al.*, 1999 ▸; Van Horn & Salpeter, 1967 ▸). The discrimination between (2) and (3) for large spheres is a matter of a third parameter, the coherence: either the reflections at the surface interfere as waves or they happen independently in the sense of particle reflections.

Typical values of κ and *k*_c_ for different small-angle scattering methods are given in Table 1 ▸. For light, recall that the contrast Δρ = π(1 − *n*^2^)/λ^2^ is related to the refractive index *n*. From these values we can see that coherent multiple scattering is most likely found for SALS followed by USANS, but coherent multiple scattering may also appear in SAXS.

## 2. A SALS example for porous materials

Frielinghaus & Gommes (2025 ▸) discussed a model function for porous materials that was originally derived for bicontinuous microemulsions (as used in the literature; Dahl *et al.*, 2024 ▸). The Teubner–Strey scattering function (Teubner & Strey, 1987 ▸) emerges from a free energy functional expansion and reads 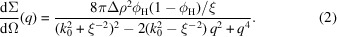
When developing surface scattering from bulk scattering, one focuses on the real-space correlation function that in the bulk case is called γ(*r*) ≃ *p*(*r*)/*r*^2^. In three dimensions, the corresponding surface correlation function is tightly connected to the square, *i.e.* γ^2^(*r*). Further details are discussed by Frieling­haus (2026 ▸). The film (surface) contrast scattering function in three dimensions then reads 
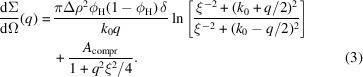
The scattering intensity dΣ/dΩ is calibrated to absolute units (cm^−1^) due to a normalization to the sample volume. The concentration ϕ_H_ refers to the volume fraction of hydrogenous material against deuterated component(s). The structural parameters of the microemulsion are its correlation length ξ and the sample wavevector *k*_0_ = 2π/*d* that connects to the domain size *d*. The last amplitude *A*_compr_ (Nallet *et al.*, 1990 ▸) emerges from the osmotic compressibility of the surfactant in the remaining fluids. [A direct link to the bulk excess surface scattering (Frank *et al.*, 2007 ▸) remains an open question. I assume a leading proportionality for the two amplitudes with large bending rigidities (Roux *et al.*, 1992 ▸; Helfrich & Servuss, 1984 ▸) and minor deviations at finite bending rigidities]. For porous materials, this term will be neglected here. Furthermore, the well calibrated amplitude of surface scattering from microemulsions is neglected for the application to coherent multiple scattering in the current example (where the contrast emerges between material and air instead of hydrogenous and deuterated materials). The correct calibrations are discussed in the original literature (Frielinghaus & Gommes, 2025 ▸).

**Figure 2:**
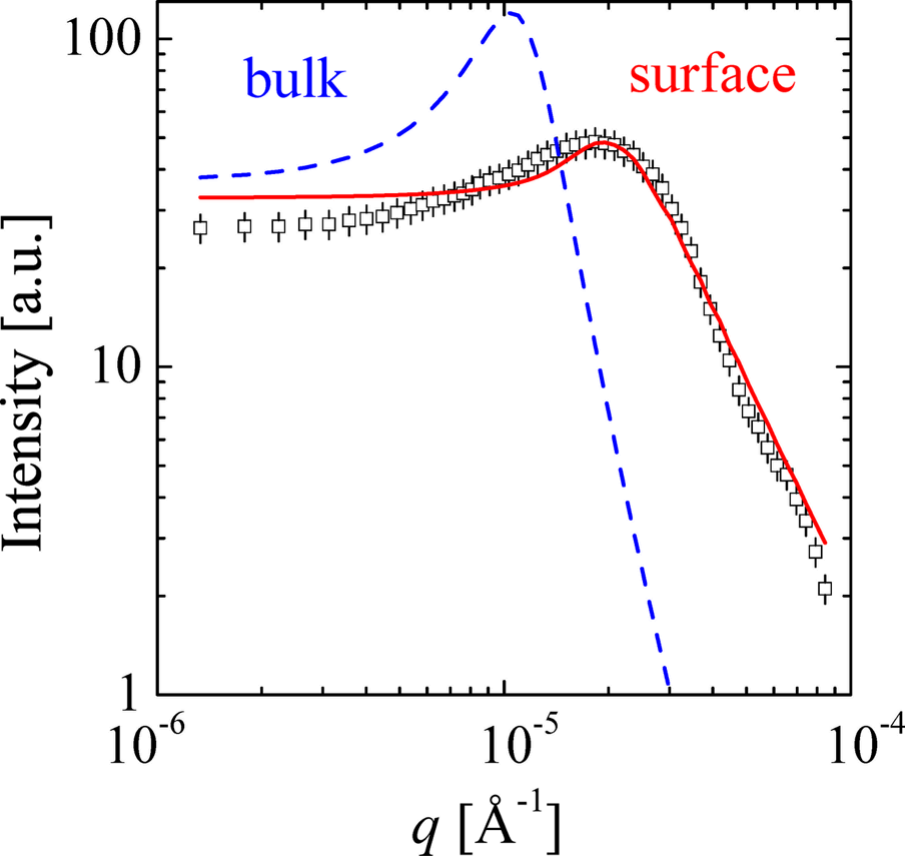
The SALS profile of a porous Al_2_O_3_ sample (black symbols), adapted from Setshedi *et al.* (2020 ▸), described by the film scattering model [equation (3)] for porous materials (continuous red line). For comparison, the corresponding bulk scattering is shown as a dashed blue line.

**Figure 3:**
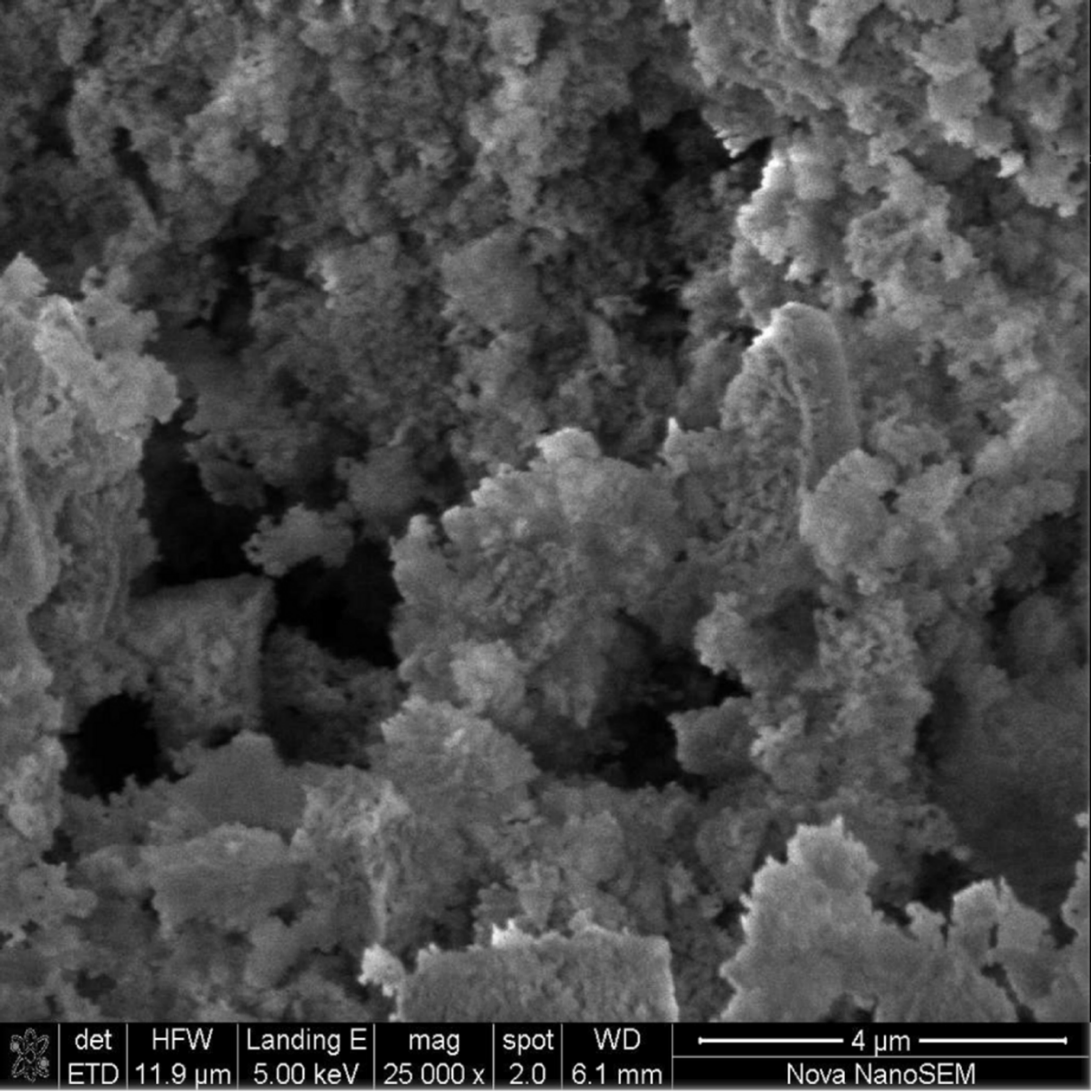
A scanning electron micrograph of the porous Al_2_O_3_ sample. Reproduced with permission from Setshedi *et al.* (2020 ▸), copyright (2020) Institute of Physics. However, the selected length scales do not fully agree with the SALS experiment.

A wide-*q*-range study, combining SALS, USAXS and SAXS, on solids that are partially porous is discussed by Setshedi *et al.* (2020 ▸). One SALS example from Al_2_O_3_ will be discussed here (Fig. 2 ▸). The critical scattering vectors are 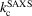 ≃ 2 × 10^−4^ Å^−1^ for X-rays and 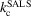 ≃ 5 × 10^−3^ Å^−1^ for light. Therefore, the whole *q* range of the SALS measurements is governed by coherent multiple scattering effects, while for X-rays the transition to power laws with smaller exponents is clearly visible for all four materials (silicon, SiO_2_, Al_2_O_3_ and TiO_2_) around the predicted 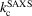. For the application of surface scattering [equation (3)], the sample Al_2_O_3_ seems to be the best choice. The model, including a resolution function and only two structural parameters ξ and *k*_0_, does not perfectly describe the peak, however, and the transition from microemulsions to porous powder samples is a big step. Nonetheless, the high-*q* power law *q*^−2^ for *q* > 3 × 10^−5^ Å^−1^ is well captured by the model. Looking at the scanning electron micrograph (Fig. 3 ▸), one can confirm that pores have evolved in the sample, although the selected size range for the micrograph seems to be too low to cover the preferred porosity in the range of ∼60 µm.

The fact that *k*_c_ for SALS is widely above the maximum scattering vector magnitude *q* causes sudden changes in the slopes of the stitched scattering curves, visible in the original publication for monocrystalline Si (Setshedi *et al.*, 2020 ▸). Other examples display slopes of −3 around the critical *k*_c_ that give rise to only small changes in the slope between mass and surface fractals. In any case, I state it is important to lower the contrast in SALS experiments when scattering profiles are stitched to SAXS or SANS curves.

A way to avoid or reduce multiple scattering is the reduction of the contrast given by |Δρ|. For neutrons, the degree of deuteration might be adapted. For SALS, the air around a solid might be replaced by solvent or the solvent in dispersions might be changed (Crosby *et al.*, 1996 ▸). Because the chemistry is changed severely by different solvents this might only apply to solids. There are other reasons (July *et al.*, 2011 ▸) (chemical hazard or monetary costs) that limit the range of contrast reduction. In summary, for neutrons a change of contrast is achieved by isotope exchange, while for other probes the chemistry of the system needs to be changed, which might affect the structure.

In the original publication (Frielinghaus & Gommes, 2025 ▸), an attempt at describing a smooth transition between surface and bulk scattering was given. A simple switching function, mainly in the region of power laws at high *q*, gave a good description (displayed there in the case of office paper scattering). The switching on a double logarithmic scale at *k*_c_ is rather sudden and happens within a few tens of per cent. Another topic is the simultaneous appearance of incoherent and coherent multiple scattering (also discussed with the same example). Usually, the incoherent multiple scattering is removed first (Jaksch *et al.*, 2021 ▸). The coherent multiple scattering can then be analysed in more detail, usually with scattering models developed independently and theoretically for surface scattering. A general strategy for removing the effect of coherent multiple scattering cannot be given for arbitrary morphologies.

## 3. Conclusions

In this paper, the important results of the full theory for coherent multiple scattering are summarized. The conditions for that case appear when turning to smaller scattering vector magnitudes *q* < *k*_c_ where the largest structures display only surface scattering. This surface scattering in the far field is a unique footpint of coherent multiple scattering and has not been described before (to the best knowledge of the author). Analytical models of simple colloidal structures (Pedersen, 1997 ▸) result in analytical formulas that can be equally well calculated for the surface structure (Ruckdeschel *et al.*, 2016 ▸; Wuttke, 2021 ▸; Volkov & Svergun, 2003 ▸). For porous structures, a recent publication (Frielinghaus, 2026 ▸) makes a connection between the bulk and surface scattering as well.

In more complex examples, a Gaussian random fields approach may provide the desired scattering functions (Gommes *et al.*, 2021 ▸). For fractal structures, the scattering exponent −α bends over to α − 6. In this paper, another example is shown for porous materials that could be connected to the classical Teubner–Strey theory. So, I expect and hope that more examples for multiple coherent scattering may be found in the near future.

## Figures and Tables

**Table 1:** Typical values of small-angle scattering experiments under different conditions For neutrons and X-rays, rather strong contrasts are assumed. For SALS, different contrast conditions are assumed.

Method	*q* (Å^−1^)	|Δρ| (Å^−2^)	λ (Å)	ξ (Å)	|κ|	*k*_c_ (Å^−1^)
SANS	10^−3^ to 0.5	6 × 10^−6^	5	50	2 × 10^−4^	10^−4^
USANS	10^−5^ to 10^−3^	6 × 10^−6^	4	5 × 10^4^	0.2	10^−4^
SAXS	5 × 10^−3^ to 1	10^−6^ to 5 × 10^−5^	1	50	10^−5^ to 5 × 10^−4^	(0.04–2) × 10^−4^
USAXS	10^−5^ to 10^−2^	10^−6^ to 5 × 10^−5^	1	5 × 10^3^	10^−3^ to 5 × 10^−2^	(0.04–2) × 10^−4^
SALS	10^−6^ to 10^−4^	3 × 10^−7^	5000	6 × 10^5^	100	5 × 10^−3^
SALS	10^−6^ to 10^−4^	10^−9^	5000	6 × 10^5^	0.5	2 × 10^−5^
